# Complications and salvage options after laser lithotripsy for a vesical calculus in a tetraplegic patient: a case report

**DOI:** 10.1186/s13037-014-0052-3

**Published:** 2015-01-23

**Authors:** Subramanian Vaidyanathan, Gurpreet Singh, Fahed Selmi, Peter L Hughes, Bakul M Soni, Tun Oo

**Affiliations:** Regional Spinal Injuries Centre, Southport and Formby District General Hospital, Town Lane, Southport, PR8 6PN UK; Department of Urology, Southport and Formby District General Hospital, Town Lane, Southport, PR8 6PN UK; Department of Radiology, Southport and Formby District General Hospital, Town Lane, Southport, PR8 6PN UK

**Keywords:** Spinal cord injury, Tetraplegia, Neuropathic urinary bladder, Vesical calculus, Laser lithotripsy, Complications, Bladder perforation

## Abstract

**Background:**

Laser lithotripsy of vesical calculi in tetraplegic subjects with long-term urinary catheters is fraught with complications because of bladder wall oedema, infection, fragile urothelium, bladder spasms, and autonomic dysreflexia. Severe haematuria should be anticipated; failure to institute measures to minimise bleeding and prevent clot retention can be catastrophic. We present an illustrative case.

**Case presentation:**

A tetraplegic patient underwent laser lithotripsy of vesical stone under general anaesthesia. During lithotripsy, severe bladder spasms and consequent rise in blood pressure occurred. Bleeding continued post-operatively resulting in clot retention. CT revealed clots within distended but intact bladder. Clots were sucked out and continuous bladder irrigation was commenced. Bleeding persisted; patient developed repeated clot retention. Cystoscopy was performed to remove clots. Patient developed abdominal distension. Bladder rupture was suspected; bed-side ultrasound scan revealed diffuse small bowel dilatation with mild peritoneal effusion; under-filled bladder containing small clot. Patient developed massive abdominal distension and ileus. Two days later, CT with oral positive contrast revealed intra-peritoneal haematoma at the dome of bladder with perforation at the site of haematoma. Free fluid was noted within the peritoneal cavity. This patient was managed by gastric drainage and intravenous fluids. Patient's condition improved gradually with urethral catheter drainage. Follow-up CT revealed resolution of bladder rupture, perivesical haematoma, and intra-peritoneal free fluid.

**Conclusion:**

If bleeding occurs, bladder irrigation should be commenced immediately after surgery to prevent clot retention. When bladder rupture is suspected, CT of abdomen should be done instead of ultrasound scan, which may not reveal bladder perforation. It is debatable whether laparotomy and repair of bladder rupture is preferable to nonoperative management in tetraplegics. Anti-muscarinic drugs should be prescribed prior to lithotripsy to control bladder spasms; aspirin and ibuprofen should be omitted. If significant bleeding occurs during lithotripsy, procedure should be stopped and rescheduled. Percutaneous cystolithotripsy using a wide channel could be quicker to clear stones, as larger fragments could be retrieved; lesser stimulant for triggering autonomic dysreflexia, as it avoids urethral manipulation. But in patients with small, contracted bladder, and protuberant abdomen, percutaneous access to urinary bladder may be difficult and can result in injury to bowels.

## Background

Laser lithotripsy of vesical calculus is considered to be a safe procedure in able-bodied individuals. We wish to highlight clinical scenario which is likely to occur in patients with spinal cord injury and neuropathic urinary bladder. Tetraplegic patients with long-term indwelling urinary catheter may have small capacity bladder; bladder mucosa may be inflamed, oedematous, and hyperaemic. Distension of the bladder during lithotripsy may result in considerable bleeding from inflamed mucosa. Tetraplegic patients may develop severe bladder spasms during laser lithotripsy of vesical calculus. Bladder spasms increase the chances of local trauma due to lithotripsy and bleeding. Bladder spasms can be reduced if spinal anaesthesia is given instead of general anaesthesia.

Spinal cord injury patients with lesion above T-6 are at risk for developing autonomic dysreflexia as a result of cystoscopy and lithotripsy. Autonomic dysreflexia is associated with rise in blood pressure, which in turn predisposes to bleeding from inflamed bladder mucosa. Urine is likely to yield growth of bacteria (commonly, coliforms) in tetraplegic patients with indwelling urinary catheters. During or after cystoscopy and laser lithotripsy, these patients may develop bacteraemia and septicaemia unless appropriate antibiotic is administered immediately before cystoscopy and continued for 48 hours.

Neuropathic bladder is often inflamed due to chronic infection and long-term indwelling catheter; therefore, the power and total energy used during laser lithotripsy should be considerably less as compared to able bodied individuals with pristine bladder mucosa. Use of relatively higher power setting and larger total energy during laser lithotripsy is likely to result in increased bleeding from the inflamed bladder mucosa and may even cause significant local injury to bladder wall. Spinal cord injury patients may be at risk for perforation of bladder wall during laser lithotripsy unless the setting for laser (power and total energy used) are kept at a lower level than what is commonly used in able-bodied individuals with healthy bladder mucosa.

Neuropathic bladder is at risk for rupture when the bladder is over-distended either due to blocked catheter or clot retention. Further, mechanical irrigation to break clots and to unblock the catheter could result in high intravesical pressures leading to rupture of a neuropathic bladder.

We report a tetraplegic patient, who developed life-threatening complications of bleeding, clot-retention and rupture of urinary bladder following laser lithotripsy of vesical calculus. We wish to share with the readers the lessons we learnt from this clinical event.

## Case presentation

**Figure 1:**
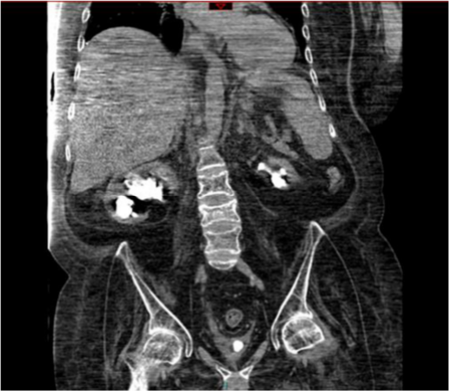
**Coronal section of CT performed prior to lithotripsy: Both kidneys normal in size; bilateral staghorn calculi larger on the right side; no hydronephrosis; empty urinary bladder with bladder stone measuring 1.5 cm in diameter.**

A British Caucasian male sustained complete spinal cord injury at C-4 in 1991 due to motor vehicle accident, when he was nineteen years old. He had tracheostomy and was ventilator-dependent. This patient underwent implantation of phrenic pacer for diaphragmatic breathing. This patient was managing his bladder by indwelling urethral catheter. He was doing well but developed severe autonomic dysreflexia due to blocked urinary catheter. This patient developed hypoxic brain injury as a result of autonomic dysreflexia. This patient had percutaneous gastrostomy. His parents were very caring and looked after him in a meticulous way. This patient developed stones in both kidneys and urinary bladder. Non-contrast CT of kidneys and urinary bladder (without contrast administration) revealed both kidneys normal in size; bilateral staghorn calculi predominantly on the left side; there was no hydronephrosis. Right lower pole renal cyst, 4.2 × 2.9 cm in size, was noted. Right mid-ureteric calculi measuring 1.7 cm in size with moderate right hydroureter was present. Empty bladder contained a stone of 1.5 cm in diameter. (Figure 1) Gallstones were present. Liver, pancreas, spleen and both adrenal glands appeared normal. No retroperitoneal or pelvic lymphadenopathy. No peritoneal effusion. Blood urea was 3.1 mmol/L. Creatinine: 19 micromol/L.

After taking written consent from parents of this patient, laser lithotripsy of vesical calculus was performed under inhalational anaesthesia. Blood pressure was 100/50 mm Hg. Laser lithotripter Slimline probe E 2 550 was used. The settings were: Pulse energy: 1.0; Total energy: 1.73 kJ; Rate (Pulse frequency): 15 pulses per second; Power level: 15 W.

Bladder was very inflamed and small capacity. During lithotripsy, patient developed persistent, severe bladder spasms and significant bleeding. Blood Pressure increased to 140/90 mm Hg during lithotripsy. After laser lithotripsy, some residual stone fragments were present. A 16 French Foley catheter was inserted; blood stained urine was being drained through the catheter.

**Figure 2:**
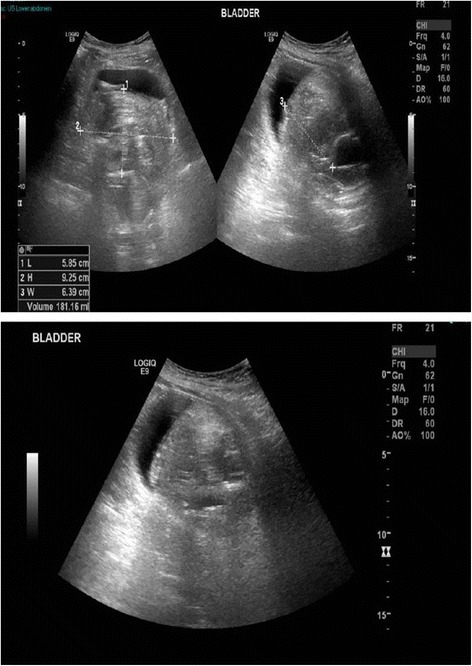
**Ultrasound scan of urinary bladder, performed seven days after vesical lithotripsy: Top panel: a large bladder clot measuring 6 ×** **9.2** **×** **6.4 cm in size, volume 181mls was present.** The bladder catheter was in situ. Bottom panel: The outline of urinary bladder was intact; there was no evidence of discontinuity of bladder wall to suggest rupture.

Following transfer to spinal intensive treatment unit, this patient continued to pass blood-stained urine but, there was no blockage of catheter. He was kept well hydrated to facilitate diuresis. However, following a weekend (four days after laser lithotripsy), a hard mass became palpable in the suprapubic region. A 24 French Foley catheter was inserted and clots were aspirated. The size of suprapubic mass decreased but it was still palpable. A 22 French three-way Foley catheter was inserted and continuous irrigation with 0.9% sodium chloride was started. He was prescribed Teicoplanin and Tazocin intravenously. Three units of red cells were transfused. CT of abdomen (without contrast administration) was performed five days after lithotripsy; this revealed 8 × 6.6 × 5.6 cm area of high attenuation within slightly distended urinary bladder, which was due to clot within the urinary bladder. There was a small residual fragment of bladder calculus measures 7 mm (previously 17 mm) and 0.9 cm calculus in the prostatic urethra (previously two prostatic urethra calculi measuring 1.2 cm and 1.3 cm). Unchanged large calculus in the right proximal ureter and unchanged multiple bilateral large renal calculi. No other significant interval change. Patient’s general condition remained unstable; Haemoglobin decreased from 102 g/L to 66 g/L. Blood pressure decreased to 79/47 mm Hg; Oxygen saturation dropped to 92%. He was resuscitated with intravenous fluids and red blood cells transfusion. Seven days after lithotripsy, bleeding continued. Ultrasound examination revealed significant bilateral hydronephrosis and hydroureter with multiple large pelvicalyceal stones. There was a large bladder clot measuring 6 × 9.2 × 6.4 cm in size, volume 181 mls. (Figure 2) The bladder catheter was in situ. No pelvic effusion was seen. A 24 French Foley catheter was inserted. Clots were sucked out. After lot of suction and irrigation, returning fluid was only slightly tinged with blood. A three way catheter was inserted and continuous bladder irrigation was commenced. Patient received four units of red cell transfusion. Patient was passing blood clots despite continuous bladder irrigation. The antibiotic was changed to Meropenem intravenously. The catheter got blocked, flushing was unsuccessful. The three way catheter was removed and another three way catheter was inserted. Bladder was washed out of clots. The catheter started to drain. Haematuria improved but continuous bladder irrigation was maintained at a slow rate. However, the following day, bleeding recurred. Blood pressure dropped to 75/40 mm Hg. Three units of red blood cells were transfused. The three way catheter was blocking frequently. Plan was cystoscopy and clot evacuation; it would be preferable to avoid open cystotomy for removing clots.

**Figure 3:**
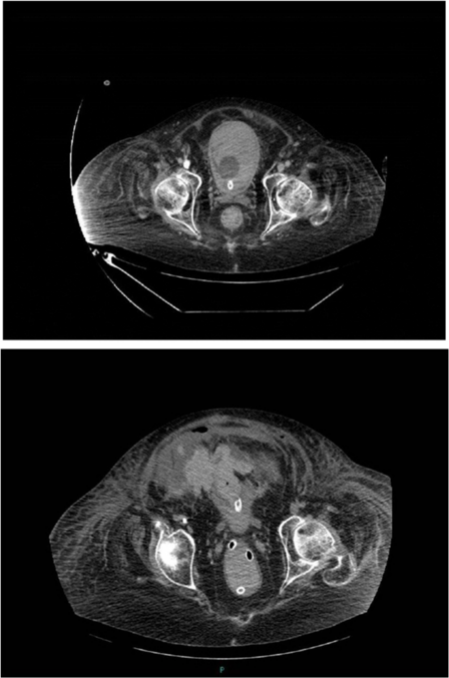
**Top panel: Axial section of CT of abdomen performed five days after vesical lithotripsy: A 8** **× 6.6** **× 5.6 cm area of high attenuation within slightly distended urinary bladder was seen, which represented clots within the urinary bladder.** The outline of urinary bladder was intact. Urinary catheter was in situ. Bottom panel: Axial section of CT abdomen with oral positive contrast, performed 12 days after vesical lithotripsy (two days after cystoscopy and clot evacuation) when the patient developed abdominal distension: A large 9 × 8 × 4.5 cm intra-peritoneal haematoma, at the superior aspect of dome of urinary bladder. There was 2.5 × 2 cm defect/perforation of the urinary bladder wall superiorly at the site of the haematoma).

**Figure 4:**
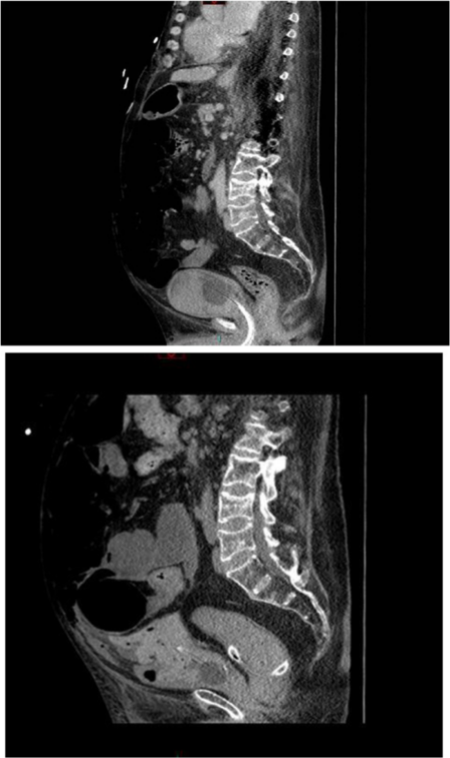
**Top panel: Sagittal section of CT of abdomen performed five days after vesical lithotripsy showed urinary bladder distended with clots.** But the outline of urinary bladder was intact. Bottom panel: Sagittal section of CT abdomen with oral positive contrast, performed 12 days after vesical lithotripsy (two days after cystoscopy and clot evacuation) when the patient developed abdominal distension: A large intra-peritoneal haematoma, at the superior aspect of dome of urinary bladder was seen which was in continuity with the lumen of the bladder. The tip of Foley catheter was abutting on the haematoma.

**Figure 5:**
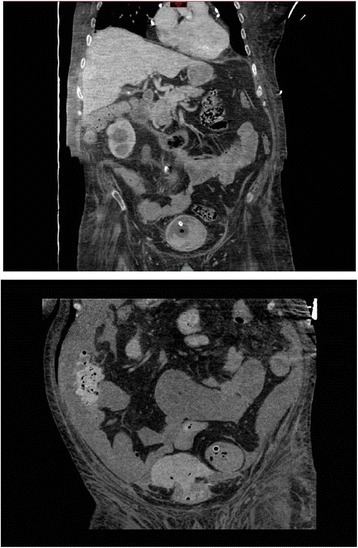
**Top panel: Coronal section of CT of abdomen performed five days after vesical lithotripsy showed urinary bladder almost filled with clots.** The outline of urinary bladder was intact. Bottom panel: Sagittal section of CT abdomen with oral positive contrast, performed 12 days after vesical lithotripsy (two days after cystoscopy and clot evacuation) when the patient developed abdominal distension: A large intra-peritoneal haematoma, at the superior aspect of dome of urinary bladder was seen with gas locules. There was a defect in the urinary bladder wall superiorly, at the site of the haematoma, indicative of bladder rupture/perforation.

Cystoscopy was performed with a 26 French cystoscope. Bladder was full of clots. There was generalised oozing. Clots were mechanically broken and majority of clots were evacuated. A 24 French three-way Foley catheter was inserted and irrigation with 0.9% sodium chloride was commenced. Patient was transferred to Intensive Treatment Unit. Abdomen became distended. White cell count increased from 5.7 to 33.8; Neutrophils increased from 3.7 to 31.9. Intra-abdominal sepsis and paralytic ileus due to perforation of urinary bladder was suspected. The dose of Meropenem was increased to 2 grams every eight hours. Two days after cystoscopy and clot evacuation, there was huge distension of abdomen. Continuous bladder irrigation was being maintained. It was not very clear whether the urine output had decreased. Bladder rupture leading to urine leak into abdominal cavity was suspected. Therefore, CT of abdomen was performed with oral positive contrast. This revealed large 9 × 8 × 4.5 cm intra-peritoneal haematoma, at the superior aspect of dome of urinary bladder, with multiple gas locules. There was 2.5 × 2 cm perforation of the urinary bladder wall superiorly at the site of the haematoma. (Figures 3, 4 and 5) Urinary catheter was in situ. Large volume of free fluid was present within the peritoneal cavity. The oral contrast had reached up to the proximal jejunum. Dilated fluid-filled loops of small bowel measure up to 4 cm were present but no discrete transition point was identified. No gross large bowel abnormality. Flatus tube was in situ in rectum. Small amount of intra-peritoneal gas and gas in the small bowel mesentery was seen in the central abdomen. No porto-venous gas. No gas in superior mesentery artery or vein. There was subcutaneous gas seen tracking along the right lateral abdominal wall extending to the right lateral chest. No pneumothorax. New extensive subcutaneous abdominal wall fat stranding noted. Bilateral small basal pleural effusions present. As before, large bilateral renal and ureter calculi noted. No other significant interval changes.

Flexible sigmoidoscopy was performed to rule out any lesion producing bowel obstruction. Sigmoidoscopy revealed normal mucosa; no colitis. A guide wire was passed and flatus tube was introduced over guide wire.

CT revealed rupture of dome of urinary bladder and intra-peritoneal leakage of fluid (urine). Further, there was no other pathology to explain intestinal obstruction. Therefore, it was concluded that the patient had paralytic ileus secondary to sepsis and intra-peritoneal leakage of urine from ruptured urinary bladder.

When the patient developed abdominal distension following cystoscopy and clot evacuation, he was transferred to intensive care unit. Meropenem and Teicoplanin were prescribed. Percutaneous gastrostomy tube was connected to free drainage. Total parenteral nutrition was administered via a central venous catheter inserted in right subclavian vein. Decision was taken against laparotomy or surgical drainage of intra-peritoneal fluid, as the intra-peritoneal fluid was thought to be urine mixed with sterile 0.9% sodium chloride used for continuous bladder irrigation.

Patient stayed in intensive care unit for 21 days. This patient developed seizures, and was prescribed levetiracetam. The tip of central line yielded growth of methicillin-resistant Staphylococcus aureus. This patient received Doxycycline 100 mg twice a day and Fusidic acid 500 mg three times a day. Patient was discharged home ten weeks after vesical lithotripsy.

**Figure 6:**
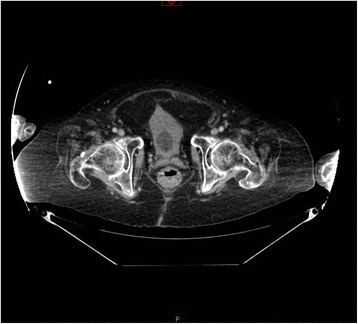
**Follow-up non-contrast CT of abdomen, performed three months after cystoscopic clot evacuation: Axial section shows intact bladder outline; no perivesical haematoma.**

**Figure 7:**
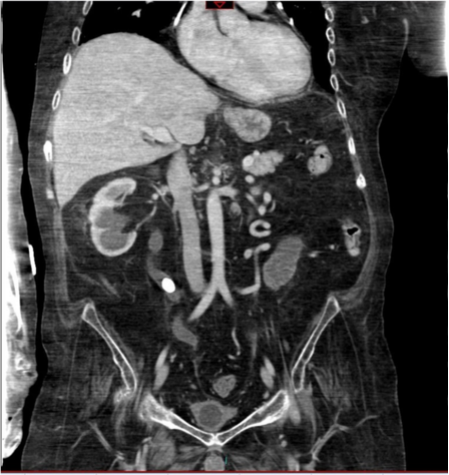
**Follow-up CT, performed three months after cystoscopic clot evacuation: Coronal section shows complete resolution of bladder haematoma and bladder perforation.**

Non-contrast CT of abdomen was performed three months later. This CT revealed resolution of the previously seen large amount of intraperitoneal free fluid, perivesical haemorrhage, abdominal wall oedema, and subcutaneous gas in right costal margin region. (Figures 6 and 7) There was no CT evidence of large or small bowel obstruction.

## Discussion

Laser lithotripsy is generally considered to be safe. We stopped using electrohydraulic lithotripsy and started using laser routinely to treat stones in urinary tract. Rarely, complications may occur, as happened in this case. Bladder perforation due to laser has been documented. Althunayan and associates [1] searched The Manufacturer and User Facility Device Experience database of the United States Food and Drug Administration and the Rockwell Laser Industries Laser Accident Database for adverse events. Overall, there were 36 (8.3%) adverse events resulting in patient harm, including 7 (1.6%) mortalities, 3 deaths from ureteral perforation using the holmium:yttrium aluminum garnet (Ho:YAG) laser, and 4 deaths from air emboli using the neodymium-doped yttrium aluminum garnet (Nd:YAG) laser. Other reported patient injuries included bladder perforation resulting in urinary diversion in a patient. Farag and associates [2] reported the first case of laser bladder perforation as a complication arising from photoselective vaporization of the prostate. This patient developed acute hyponatremia, rhabdomyolysis and acute renal failure.

In our patient, the probable sequence of events is given below:

Small capacity bladder predisposed to bladder spasms when lithotripsy was performed under general anaesthesia. Significant bleeding occurred from the inflamed bladder mucosa, compounded by rise in blood pressure as a result of autonomic dysreflexia and severe local trauma due to use of laser in the bladder undergoing repeated spasms. Continued bleeding led to clot retention and over-distension of bladder. Although clots were sucked out, continued bleeding because of local tissue injury caused by laser, resulted in repeated clot retention and over-distension of inflamed, neuropathic bladder. Over-distension of bladder due to clot retention, rigid cystoscopy, and mechanical irrigation to suck out clots paved the way for bladder rupture.

Rupture of urinary bladder following cystoscopic clot evacuation has been reported. Smith and associates [3] described two cases of rupture of the urinary bladder following cystoscopic clot evacuation. Both patients had hemorrhagic cystitis secondary to cyclophosphamide therapy. The bladder injuries were not immediately recognized. Computed tomography demonstrated the bladder rupture in both patients. Pre-disposing pathologies for bladder rupture are increased intra-vesical pressure and a decrease in the strength of the bladder wall. [4] Over-distension of bladder due to clot retention increases intravesical pressure and weakens the neuropathic urinary bladder wall, which was already fragile due to chronic infection in this tetraplegic patient.

Continuous bladder irrigation was carried out in this patient to prevent clot formation; in addition to continuous bladder irrigation, intermittent mechanical irrigation was performed to dislodge clots which were blocking the catheter, and to remove clots from the urinary bladder. Continuous bladder irrigation allows blood from the urinary bladder to be immediately evacuated, thus preventing clots from forming. The goal of this treatment is to prevent the need for surgical intervention by continuously flushing out clots, while the bleeding area heals. Inflow of irrigating fluid should be done via low gravity, and not forced into the bladder with pumps or pressure. This allows the inflow of irrigant to stop immediately if the outflow is compromised or clotted. If the fluid is forced into the bladder with an infusion pump or manual irrigation while the outflow is clotted, bladder may rupture. This patient received manual irrigation to unblock the catheter and to remove clots while the patient was staying in intensive treatment unit and in the operation theatre where cystoscopic clot evacuation was carried out. It is possible that bladder rupture occurred during mechanical irrigation of bladder by Ellik evacuator or using a catheter-tip syringe, or during instrumentation.

Manley and associates [5] reported a patient, who suffered iatrogenic intra-peritoneal bladder rupture while being administered continuous bladder irrigation by health professionals using an infusion pump instead of instilling the irrigation via low gravity. Twenty-four hours later the patient developed a bladder rupture that was attributed to the high pressure with which the irrigation fluid was infused.

Treatment of rupture of urinary bladder is usually surgical. But conservative therapy is preferable in patients with other serious co-morbidities as in our patient with tetraplegia. Hagiwara and associates [6] reported rupture of neurogenic bladder in a 44-year-old woman. She was cured by conservative therapy, including catheter drainage and antibacterial chemotherapy. Leyland and associates [7] reported conservative management of an adult who had undergone augmentation cystoplasty and continent urinary diversion, and developed intra-peritoneal rupture of bladder. The present authors had experience in conservative management of bladder rupture of varied aetiology in eight patients. [8] Criteria of patient selection for this mode of management were important for successful outcome. Seven out of eight patients were cured completely; cause of death in one patient was not related to bladder injury or its complication.

Choice of anaesthesia in a tetraplegic patient is controvertial. We have used spinal anaesthesia to control bladder spasms and autonomic dysreflexia. [9] However, spinal anaesthesia in a patient with spinal cord injury, who is already predisposed to autonomic dysreflexia, could be more risky as there could be sudden shifts in vascular compartment leading to hypotension, thereby precipitating crisis. If at all spinal anaesthesia is to be used, isobaric agent should be used to control the level of block. Availability of isobaric agents for spinal use might be an issue as they may not be routinely available in many centres.

Similarly, choice of surgical procedure for removal of stones from urinary bladder in tetraplegic subjects is debatable. Percutaneous cystolithotripsy using a wide channel could be less traumatic, quicker to clear the stone as larger fragments could be retrieved, lesser stimulant for triggering autonomous dysreflexia, as it avoids urethral manipulation. In fact cystolithotomy using a small incision with rapid clearance of stone would merit consideration in a tetraplegic patient with several co-morbidities. However, it should be borne in mind that percutaneous access to urinary bladder may be very difficult in tetraplegic patients with protuberant abdomen, spinal curvature, small and contracted urinary bladder; injury to bowels may occur during blind percutaneous access to urinary bladder in spinal cord injury patients.

## Conclusion and take-home message

Anti-muscarinic drug therapy should be prescribed prior to lithotripsy to control bladder spasms. Drugs, which may increase bleeding during lithotripsy (aspirin, low-molecular weight heparin, non-steroidal anti-inflammatory drugs) should be omitted prior to lithotripsy. If significant bleeding occurs during lithotripsy, the procedure should be stopped and patient booked for second session of lithotripsy for removing residual stone fragments after two to three weeks. Three way catheter should be inserted and continuous bladder irrigation should be started if there is significant bleeding following vesical lithotripsy. It is preferable to prevent clot retention right from the beginning. Caution should be observed while unblocking the catheter or removing clots from the urinary bladder in spinal cord injury patients with neuropathic bladder. While using Ellik evacuator or catheter-tip syringe, high intravesical pressures may be generated unwittingly. Even transient increase in intravesical pressure may lead to rupture of a distended bladder, which is already diseased due to spinal cord injury.

## Consent

Written informed consent was obtained from the parents of this tetraplegic patient with brain injury, who was unable to give consent, for publication of this Case report and any accompanying images. A copy of the written consent is available for review by the Editor-in-Chief of this journal.
